# The background and legacy of Lewontin's apportionment of human genetic diversity

**DOI:** 10.1098/rstb.2020.0406

**Published:** 2022-06-06

**Authors:** John Novembre

**Affiliations:** ^1^ Department of Human Genetics, University of Chicago, Chicago, 60637, IL; ^2^ Department of Ecology and Evolution, University of Chicago, Chicago, 60637, IL

**Keywords:** human, population structure, human genetic diversity, genetics and race

## Abstract

Lewontin's 1972 article ‘The apportionment of human diversity’ described a key feature of human genetic diversity that would have profound impacts on conversations regarding genetics and race: the typical genetic locus varies much less between classical human race groupings than one might infer from inspecting the features historically used to define those races, like skin pigmentation. From this, Lewontin concluded: ‘Human racial classification … is now seen to be of virtually no genetic or taxonomic significance’ (p. 397). Here, 50 years after the paper's publication, the goal is to understand the origins and legacy of the paper. Aided by insights from published papers and interviews with several of Lewontin's contemporaries, I review the 1972 paper, asking about the intellectual background that led to the publication of the paper, the development of its impact, the critiques of the work and the work's application and limitations today. The hope is that by gaining a clearer understanding of the origin and reasoning of the paper, we might dispel various confusions about the result and sharpen an understanding of the enduring value and insight the result provides.

This article is part of the theme issue ‘Celebrating 50 years since Lewontin's apportionment of human diversity’.

## 1. Introduction

And so I thought, ‘Well, we've got enough of this data, let's see what it tells us about the differences between human groups’. And so I just looked into the literature, and that literature was in books and so on.

[O]ne day I was going to give a lecture, I think it was in Carbondale, Illinois, or somewhere south. I was working in Chicago at the time. So I took a couple of these books with me and a pad of paper, and a table of logarithms which I needed for this purpose, and a little hand calculator, and I sat on this bus trip for three or four hours looking at the books, picking out the data, looking it up in the table of logarithms, doing a calculation, and writing it down in tables. And when I got back after the round trip I had all the data I needed to write the paper about how much human genetic variation there was, and so I did it. […]

Shows you it's worthwhile being afraid to fly, by the way, because you have lots of time on a bus to work. – R.C. Lewontin, Interview for ‘Race the Power of an Illusion’ Documentary [1]

With this story, Lewontin describes the origins of his paper on ‘The apportionment of human diversity’ [2], a paper that would go on to have profound impacts on perceptions of human genetics and race, with key phrases from the paper echoing for decades across disciplines to the present day.

As is well known, the paper showed that at a typical genetic locus 85% of ‘[genetic variation is found within human groups]' and on that basis he concluded that ‘human races and populations are remarkably similar to each other with the largest part by far of human variation being accounted for by the differences between individuals’ and from this he judged that ‘Human racial classification is now seen to be of virtually no genetic or taxonomic significance’. The paper's main result is routinely cited in antiracist perspectives on race and genetics and has been featured in some of the most widely distributed public translations of genetic results (e.g. [1,3]).

Here, 50 years after the paper's publication, the goal is to understand its origins and legacy, including the nature of the critiques and concerns with it, and ultimately what power and limitations it has in discussions about genetics and race. Aided by insights from published papers and interviews with several of Lewontin's contemporaries (see Acknowledgements), I review the 1972 paper, asking about the intellectual background that led to the publication of the paper, the development of its impact, the critiques of the work and its enduring relevance. This review also benefits greatly from insights provided by other perspectives on Lewontin's 1972 [2] paper (e.g. [4–6]), including an edited volume on the papers of Anthony Edwards [7], which contains an excellent interview with Edwards and an especially lucid commentary by Noah Rosenberg [8].

The next section of this review, ‘Background’, situates Lewontin's work in terms of a complex set of antecedents, including his now little-known work on the textbook *Quantitative Zoology* [9], as well as an interest in information theory and debates in evolutionary genetics at the time. The hope is that by understanding the foundations of the work, we might gain a deeper understanding of the reasoning of the paper, and in turn more clearly navigate the subsequent critiques.

The third section, ‘The results and reactions', discusses how his key result was both supported and contested by subsequent publications. Many of the critiques criticized a conclusion that Lewontin did not in fact draw; the apparent critique by Edwards [10] in particular considers a different aspect of the taxonomy problem from the one Lewontin aimed for and does not invalidate the central insight provided by Lewontin's result. We will see how much of the apparent controversy here was due to a confusion about the focal question. For Lewontin, the question was not whether genetics can be used for studying relationships among individuals (i.e. whether genetics can be a basis of taxonomy); he was asking rather whether human racial groupings have taxonomic significance in the sense that they are predictive of meaningful differentiation at a *typical* genetic locus.

The fourth section, ‘Lasting legacy’, considers how the Apportionment's key result became a sound bite, so well-used that it is at times applied in vague and mistaken ways even by its advocates, a slippage that likely invited some of the later critiques. Despite its importance, Lewontin understood that the result does not provide a single answer to all arguments about genetics and race. Yet, in showing how human genetic differentiation is much lower than one would suppose from looking at surface indicators like skin pigmentation, the result endures as a key fact about human genetic variation that reaffirms that nothing about human genetic diversity is as simple as it first appears. Finally, box 2 on ‘Teaching the result’ gives some insights and references on some approaches to teaching the result.

## 2. Background

### (a) In the spirit of *Quantitative Zoology*?

Lewontin's paper was developed at a time when many geneticists found themselves being called upon to react to the controversial writings on genetics and race by Arthur Jensen in 1969 [30]. Lewontin himself had written a critical response in the *Bulletin of Atomic Science* already in 1970 [31] and later wrote similarly motivated pieces, for example, in *Science* in 1975 with Marcus Feldman [32], in the *American Journal of Human Genetics* in 1974 reacting to works by Morton and colleagues [33], and much later in 1984 in *Not In Our Genes* [34]. Though the Jensen controversy was likely among the motivating factors, the 1972 paper does not contain any reference to it.

The paper appears in the 6th volume of a series titled *Evolutionary Biology* for which Theodosius Dobzhansky (Lewontin's PhD advisor), Max Hecht and William Steere were editors. The volume appears to have been organized, at least in part, to honour famed paleontologist George Gaylord Simpson's 70th birthday. The opening piece is a tribute to Simpson [35], and many of the articles, though not all, begin with tributes to him, including Dobzhansky's contribution to the volume [36]. The volume also has a concentration of papers on human variation and evolution: the final five papers in the volume are focused on the subject, including a contribution by Dobzhansky, who possibly invited Lewontin to submit to the volume. One of those five human-oriented papers was titled ‘Polygenic inheritance and human intelligence’ [37], though its author, Michael Lerner, tried explicitly not to discuss the ongoing controversies about human genetics and race. Dobzhansky himself had long been steeped in discussion about the taxonomic value of the race classification, defending a concept of race in biology and its applicability to humans [38].

Whether Dobzhansky and the other editors were seeking a piece from Lewontin that might honour Simpson and/or address genetics and race in humans is unclear. Lewontin and Simpson likely interacted when they were both at Columbia in the early 1950s, and while relatively unknown today, Lewontin had a substantial collaboration with Simpson. In 1960, Simpson and Lewontin co-authored the second edition of the textbook *Quantitative Zoology*, along with Simpson's wife Anne Roe [9].

First published in 1939, the book teaches a rigorous, numerical approach to questions in biology, including taxonomy. Lewontin was added as an author for the 1960 edition due to his expertise in biometrics, a field that had blossomed since the original publication of *Quantitative Zoology*. The new edition added a chapter on the analysis of variance (‘This must now be considered an essential and basic technique in *Quantitative Zoology*' p. vi), which Lewontin most likely wrote given the style and themes that it emphasizes.

As an application of the analysis of variance, the chapter describes how ‘a problem common in experimental taxonomy is that of distinguishing geographical races' (p.304). Though it is not explicit about defining criteria for races, it presents an example from *Drosophila persimilis* from three geographical localities in western North America. Unlike what he would later find for human groups, the genetic variance in bristle number across localities was estimated to be about 4-fold larger than that between individuals. As the chapter explains ‘The reasonable conclusion from the analysis is that the populations […] are genetically different and constitute distinct geographical races (under one commonly accepted criterion of race).’ (p.304)

Though differing in detail (using indirect estimates of genetic variance in an example trait versus direct observation of genotypic data) and imprecise in defining a criterion for race, the approach foreshadows Lewontin's work on human genetic diversity in the 1972 *Apportionment* paper. Both works apportion variances to make evaluative statements about taxonomic categories. The working logic is that a meaningful taxonomic assignment (e.g. of individuals into races) is one where a large amount of variance between groups exists relative to the variance within.

Another relevant passage that is worth revisiting as background to the 1972 [2] paper, comes from the chapter on the ‘Comparison of Samples'. In it, the classic distinction between statistical significance and biological significance is made (emphasis preserved from original text):

In the real world, no two populations of objects can have precisely equal means or standard deviations. A difference between two such populations can always be established, provided the observer is willing to go to enough trouble to refine and increase the number of his measurements. (p.173, [9])

And it continues:

One always knows long before he starts that both the samples which he measures and the populations from which they are drawn are different to some greater or lesser extent in the value of every parameter that can be imagined. It is therefore essential that some degree of difference be assumed to be trivial and not germane to the problem. (p.173, [9])

Applied to genetics and put in the context of his 1972 paper on humans, the question is not whether differences in allele frequencies between named races exist—it is rather whether the differences between groups are meaningful or, as he describes in the passage, can be ‘assumed to be trivial and not germane to the problem’. As we will see, the apportionment of diversity would be Lewontin's numerical tool for addressing this question.

### (b) Evolutionary motivations?

Whether prompted by the volume dedicated to Simpson or not, Lewontin did not explicitly acknowledge any link in his 1972 paper. Lewontin's introduction to the paper and later interviews would suggest that the motivations were larger than the volume, and that any framing in terms of Simpson did not bear heavily on his mind. Lewontin introduced the paper in the context of major questions in evolutionary genetics from the time, and the challenge of how to measure variation between populations.

The late 1960s and early 1970s were a dynamic time for evolutionary genetics, when major schools of thought were being challenged by new tools for the measurement of genetic variation (namely gel electrophoresis) that were revealing an abundance of molecular variation. In one major school of thought, the ‘Classical’ school of H.J. Muller and colleagues, most genetic variation is deleterious and rare; in contrast, for the ‘Balancing’ school, associated with Dobzhansky and colleagues, genetic variation is abundant and maintained by selective forces, such as heterozygote advantage or temporally varying selective pressures [33,39]. These schools of thought were to be up-ended shortly by a new school that supposed that most molecular variations were neutral with respect to fitness (a hypothesis first raised by Lewontin in [40], p.605).

In the 1972 paper, Lewontin motivated the apportionment of diversity among races in light of these Classical and Balancing schools. For the Classical school, any differences between biological races must be a major component of the total variation in a species; while for the Balancing school, variation between races would be expected to be a small component of variation. For Lewontin, understanding the apportionment of variation in humans, or any species for that matter, had a relationship to the broadest questions of evolutionary genetics. The 1972 paper would not be remembered for those reasons, though. Even in the paper itself, Lewontin does not return to the broader evolutionary framing when discussing the implications of his result.

Lewontin also set the stage for his paper by pointing out what he saw as an epistemological problem to assessing how much variation lies between groups. If the same characters used for grouping individuals are used for assessing the partitioning of variances (e.g. skin pigmentation in the case of human races), then the magnitude of between-group variation will necessarily be over-estimated.

Thankfully for Lewontin's purposes, in the lead up to his paper, ever-broadening surveys of genetic variation in human groups were taking place (e.g. [41–45]). These direct observations of genetic variation were beginning to be analysed with a growing suite of numerical approaches, including population clustering and phylogenetic analysis techniques [46–48], and had the exciting promise of overcoming the perceptual biases that would otherwise plague apportionments of diversity in humans.

In Lewontin's words, molecular data could ‘put the comparative differentiation within and between groups on a firm quantitative basis [2, p. 383].’ This emphasis on a technological opportunity dovetails best with how he would later describe the setting for the work. For instance, as quoted above from a 2003 interview [1], Lewontin set up his 1972 paper by describing how the accumulating datasets were key to spurring his work on the problem. Lewontin appreciated that the time was ripe for carrying out a quantitative analysis of the taxonomic groupings of humans into races.

### (c) The information theory content in classification?

A methodological curiosity of the 1972 paper is that although he was well versed in analysis of variance (as described above) and having titled the paper the ‘apportionment of human diversity’, Lewontin did not use a traditional analysis of variance nor the already well-known alternative based on Sewall Wright's heterozygosity-based measures [49]. Instead, he used an approach based on Shannon's information theory [50]. Information theory was popular at the time across numerous disciplines and Lewontin reportedly took interest in it. The information-theoretic approach yields nearly identical results to using heterozygosities (as well as to using later versions of the analysis of variance modified for genetic data), but as many authors have noted, the information-theoretic approach is non-standard and more difficult to interpret in standard genetic terms [51–53].

Whether convenience played a role is amusing to consider—perhaps computing logarithms via tables was simpler at the time than the quadratic forms needed for computing heterozygosities, especially if the calculations truly took place mostly on a bus ride. In his rationale, Lewontin hints that the reasoning was more profound: ‘[Shannon's information measure, *H*] is widely used to characterize species diversity in community ecology, and since I am performing a kind of taxonomic analysis here, I will use *H*’ [2, p. 388].

With regard to his goals with the paper, in carrying out ‘a kind of taxonomic analysis' with measures of information theory, his analysis considered how much information there is among individuals within populations relative to the whole (that is the average proportion of diversity within populations was calculated as ${\bar{H}}_{\mathrm{pop}}/H_{\mathrm{species}}$). If that proportion is large, one might conclude that the difference between races is small.

Another way of conceiving the question in terms of information content is to ask whether a taxonomic label has much information about an individual's alleles (see box 1)? Does knowing two individuals' race assignments give you much information about their genetic differences? (Reference [54], for example, verbally frames the question in these terms.) The answer to these questions would seem useful for evaluating the taxonomic value of the race designation, and Lewontin's investigation would shed light on these important questions (box 2).

Box 1. **Information-theoretic interpretation of Lewontin's entropy calculations.**

Although Lewontin motivated the use of Shannon's information measure by noting its use in community ecology, his approach has an information-theoretic interpretation. Consider the problem of communicating the alleles in a particular individual's genotype to an observer by using a digital message. How many bits would be required to convey each allele, and could the message be made substantially shorter if an individual's population or race label is known?

From information theory, the average length needed to convey a random variable's value, assuming an optimal encoding scheme, is the *entropy* of the random variable. Lewontin computed the entropy at different hierarchical levels, in each case computing a variant of $H=-\sum_{i}\;p_{i}\ln_{2}p_{i}$, where the summation is over the alleles at a locus and $p_{i}$ is allele frequency at the level of analysis being considered (e.g. population, race, species). This entropy conveys the average message length for communicating one of the alleles in the genotype in an encoding scheme that ignores any correlation among alleles within and between loci. As an example, at a bi-allelic locus, 1 bit would be required for an allele with frequency 0.5. Because the species-level entropy value ($H_{\mathrm{species}}$) computed by Lewontin uses an allele frequency averaged over all sampled populations, it can be understood as the expected length to communicate an allele without using any race or population label. Following a similar logic, his entropies ${\bar{H}}_{\mathrm{pop}}$ and ${\bar{H}}_{\mathrm{race}}$ can be understood as expected lengths in coding schemes that condition on knowing the individual's population and race labels, averaged over all populations/races (this implicitly assumes that the individual is equally likely to come from any of the populations or races). Lewontin's calculation of particular ratios of entropies helps reveal the fractional reduction in message length achieved by using different schemes.

His value for ${\bar{H}}_{\mathrm{pop}}/H_{\mathrm{species}}\;$of 85.4% indicates that having population labels would reduce the message length by 14.6% on average, and the value of $(H_{\mathrm{species}}-\;{\bar{H}}_{\mathrm{race}})/H_{\mathrm{species}}$ of 6.3% indicates that having race labels would reduce the message length by 6.3% compared to using no labels. In information-theoretic terms, the salient concept is conditional entropy, which measures how much the message length decreases as we provide more information to the receiver.

Box 2. **Teaching the result.**

Part of the legacy of any major scientific result is how it is taught. The apportionment of diversity can at times be difficult for students to grapple with—it is difficult to think in terms of analysis of variance.

Thankfully, Lewontin's work can be taught to students in a number of ways because the apportionment of diversity has relationships to many other concepts in population genetics: the deficiency of heterozygotes observed in a sample, the expected number of differences between pairs of sequences, the numbers of shared versus private variants, and pairwise coalescent times [8,11–14].

Different graphical representations of the result can help. Donovan *et al.* display the result in terms of overlapping circles in a curriculum designed for high school students (fig 1 in [15]). Mountain and Ramakrishnan [16] plot pairwise differences between individuals from within and between populations (also see [17]). Rosenberg [13] has a number of different relevant and interesting figures based on micro-satellite data. Finally, many of the recent landmark papers on worldwide human population genomics have useful figures within them (e.g. fig. 1A from [18]; fig. 2 from [19]). My group also recently published an approach that visualizes the geographic abundance patterns of variants in the high-coverage 1000 Genomes dataset [18,20] to help students understand how a ‘typical’ variant is geographically distributed (Figures 1, 3, 4, [11]; also see [21]).

In my own teaching, I have developed an introductory population genetics workshop that uses real-world data on the deficiency of heterozygotes relative to Hardy-Weinberg proportions to teach about human genetic structure and how skin pigmentation loci, such as SLC24A5, are outliers relative to the typical locus [22]. In lectures, to emphasize the point of Edwards [10] and how information combines across many markers, I also enjoy showing Fig. 2 from Novembre & Peter [23] as well as Fig. 6 from Patterson *et al.* [24] and Fig. 2C from McVean [25].

Without question, more pedagogical research on the impacts of how we teach about human genetic diversity is needed. As an example, Donovan *et al.* [15,26] have carried out preliminary studies to show how at least some approaches can reduce racist attitudes among students (also see [27–29]). More quantitative research on pedagogical outcomes of genetics education of this sort would be greatly beneficial.

## 3. The result and reactions

### (a) Arbitrary choices, yet an enduring result

To carry out his analysis, Lewontin used published data [41,42,44,45] and had to make several decisions: which human groups to include, how to arrange them into various populations, and then how to organize the populations into races. This is not a straightforward exercise, and Lewontin's text is frank regarding the challenge in a way that one rarely reads today: ‘I have tried to include what would appear to be *a priori* representative of the range of human diversity. But how does one do that? […] How many different European nationalities should be included as compared with many African peoples or Indian tribes?’ (p.384). He ends up choosing to use ‘as much as possible equal numbers of African peoples, European nationalities, Oceanian populations, Asian peoples, and American Indian tribes' (p.385) weighing them equally, with the rationale that the equal weighting should favour finding variation between groups versus within them. With regards to the racial groupings, ‘the boundary line must be arbitrary’ (p.385) but ‘I have chosen a conservative path and have used the classical racial groupings with a few switches based on obvious total genetic divergence. Thus the question I asked is ‘‘How much of diversity between populations is accounted for by more or less conventional racial classification?'’’ (p.386). To that end, he uses what he calls ‘the usual four’ (i.e. ‘Caucasians’, ‘Black Africans’, ‘Mongoloids’, ‘Amerinds’), as well as ‘South Asian Aborigines', ‘Oceanians’ and ‘Australian Aborigines'.

With those designations he found his well-known results: the average proportion of diversity within populations was 85.4% of the total, between populations within races was 8.3% of the total, and a final 6.3% was accounted for by diversity between the race groups. Given the arbitrariness of how to group populations into races, the 85.4% value is expected to be the more robust than the 8.3% and 6.3% values. Later results and re-calculations show that these numbers vary depending on grouping decisions [6] as well as due to calculation errors [55]. Over time, the 85% number would be replicated with some deviations (e.g. 80–95%) across a number of studies using related approaches on similar data, including by Nei and Roychoudhury that same year and by others shortly after using similar data [51,56–60], and with novel forms of data such as alleles at HLA loci ([56,61]; also see [62]).

Over time, yet more marker systems have shown similar results, such as mitochondrial variation [63], RFLPs [64,65], microsatellites [64,66,67] and genome-wide surveys of single-nucleotide polymorphisms (SNPs) [68,69]. For example, with microsatellites, Rosenberg *et al.* [67] found the proportion within populations to be higher, at 93–95%. Using large samples of bi-allelic SNP markers, Li *et al.* [69] found a number slightly higher than Lewontin's, with 89% within populations. The biological and technical nuances affecting the differences in these numbers have been discussed previously (for example by [6,70,71]). Some of the differences may be due to Lewontin's resolving of arbitrary choices by choosing those that would favour observing between-group variance; however, some of the differences in results across studies may be due to the different loci used. Despite these variations, the qualitative finding of much higher variation within human populations relative to the variation between populations or races has been robust.

In a public talk at Berkeley years after his original publication [72], while describing how Barbujani's [6] study found 84.5% within populations as opposed to Lewontin's 85.4%, Lewontin would quip that the results were ‘closer than any scientist is entitled to expect’ and he joked that given the permutation of the last two digits ‘[Barbujani] got the last two numbers wrong’ ([72], *ca* 30:45). Barbujani told me how when he conversed with Lewontin about his paper, Lewontin was happy about the agreement they found on the roughly 85% within-population portion but was concerned about an apparent discord regarding the between-race portioning, as Barbujani's number was twice that of Lewontin's (12% versus 6%). As Lewontin expressed in the same public talk, the between-race amount comes down to ‘how you decide who goes in what race’, and stressed the 85% variation as being consistent and ‘pretty remarkable’. With the benefit of hindsight, the results of Rosenberg *et al.* [66] suggest that the similarity between Lewontin and Barbujani *et al.*'s analyses may have been in part be due to how Barbujani *et al.*’s study has one factor in the study that magnifies differentiation slightly (a curated population set with more isolated populations) and another that reduces the differentiation (the use of microsatellites).

Regardless, the implication of Lewontin's ‘pretty remarkable’ result was, as he stated in the conclusion of his 1972 paper, that ‘our perception of relative large differences between races and subgroups, as compared to the variation within these groups is a biased perception’. This conclusion has also held up remarkably well in the age of genomics. Human population geneticists now know much more about the genetic basis of skin pigmentation, and have directly observed how the large-effect genetic loci that underlie skin pigmentation are systematically more differentiated than a typical locus in the human genome [73–75]. In agreement with this, Relethford estimated the apportionment of diversity for skin colour as a trait, finding the diversity apportions with 91% of the variance *between* populations [76]. In addition, models of human population history that have been fit to genetic data overwhelmingly support that the divergences among human groups are all relatively recent in evolutionary terms and that gene flow across seemingly distinct geographical regions has been common [77–79]. That history helps explain why most of the variation is found between individuals rather than between ‘races’: for the typical locus in the human genome, there simply has not been enough time for substantial differentiation between groups to emerge, especially with gene flow acting as a homogenizing force.

The presence of genetic ancestry from archaic hominins, such as Neandertals and Denisovans, slightly complicates this picture, but given the relatively small fraction of the genome inherited from archaics, the effect is arguably minimal (see [80]). Thus, while Lewontin did not dwell on historical mechanisms in his original paper, today there is a much larger, coherent body of evidence and a set of detailed historical population models that support the remarkable observation he found.

### (b) Dimensions of confusion

While the apportionment result has been enduring, Lewontin's final interpretations in the paper have had a more complicated fate [2]. In his final paragraph, Lewontin stated how ‘human racial classification’ is ‘now seen to be of virtually no genetic or taxonomic significance’, and as a result ‘no justification can be offered for its continuance’. His critique here is of an existing classification system that groups human individuals by races (i.e. a categorization of individuals; also see Shen & Feldman [81]; also see [54]). Keeping in mind the background given above on the analysis of variance, he is giving his conclusion that the quantitative differences between races are small enough to not be biologically meaningful. In turn, race as a taxonomic label is not very meaningful or useful as a predictor.

The specific words used here turn out to be important, and Lewontin's language here became a source of confusion (again, see Shen & Feldman [81]). Many would think he was saying that using genetics to assign people to genetic populations (a different type of classification from human racial classification) is not possible at all. Among these, a critical paper by Edwards in 2003 titled ‘Human genetic diversity: Lewontin's fallacy’ is the most well known, but it was not the first such critique.

Foreshadowing Edwards, in 1976, Richard Spielman and Smouse began their paper on the ‘Multivariate classification of human populations' [82] with a critical stance toward Lewontin's work: *… it has been claimed that the variation within each human population is so great that population distributions are more notable for their overlap than for their distinctiveness. If this claim is justified, it follows that an individual cannot be confidently assigned to the correct population on the basis of phenotype. This view of human diversity is a natural outgrowth of the examination of many different characters, taken one at a time; single characters usually do show large overlap among populations. A multivariate approach to the question might well yield a different answer however, […] How reliably can an individual be placed in the correct population, if one makes use of all the information available?* Spielman and Smouse then went on to show how a multivariate/multi-locus approach allows assignment of Amazonian individuals to villages or clusters of villages better than random expectation.

In a similar spirit, Mitton [83] criticized Lewontin [2] as well as Nei and Roychoudhury's similar papers [59,60], see above). He argued that one obtains a very different partitioning of variance when shifting from a single-locus to multi-locus perspective. By looking at multiple loci jointly, Mitton found a much greater partitioning of variance among populations. Mitton's analysis motivated three separate critical responses [84–86]. When Mitton replied to their critiques, he held fast that a different perspective emerges from a multi-locus approach [87], including a principal components analysis of 92 population samples that showed three clear clusters—labelled Oceanians, American Indians and Africans. The point being, as in Smouse and Spielman's work, that individual assignment to populations is possible using multiple loci.

These same points first raised in the late 1970s would be arrived at independently by Anthony Edwards in 2003, in his impactful paper titled: ‘Human genetic diversity: Lewontin's fallacy’. Edwards argued that even with most of the variation found within populations, genetics can be used for assignment of individuals to genetic populations (a fact that had just been shown empirically by [67]). Edwards developed the point with an elegantly simple two-population mathematical model, and emphasized the information contained in the joint distribution of all the observed loci. Scientifically, his argument is similar to the points of Spielman and Smouse and Mitton, but the presentation is more pithy and has proven more impactful. Edwards was also the most polemical. He characterized Lewontin's work as ‘an unjustified assault on classification, which he deplored for social reasons'.

With regards to the legacy of Lewontin's result, one passage from Chakraborty's response to Mitton is particularly illuminating: ‘if it is possible to score the entire human genome we would be able to infer that there is a vast amount of genetic diversity even between individuals of the same family. … To have a uniform unit for all evolutionary studies [a] ‘per cistron’ unit is probably more profitable. [84, p. 1137]’ The phrasing ‘per cistron’ is antiquated but means essentially ‘per locus’ in modern parlance (i.e. per a given location in the genome). The passage highlights how—whether apportioning diversity or doing classification—a recurring challenge with these discussions is, what aspect of genetic variation is one concerned with: a single ‘typical’ locus, the entire human genome, or the variants underlying a given phenotype? As we see, dimensions of confusion emerged regarding (1) whether Lewontin was arguing that classification (in the sense of assigning individuals to populations) was possible or not, and (2) whether his focus was on genetic variation at single loci or multiple loci.

A remarkable aspect about the collection of critiques to Lewontin is that they are tangential to Lewontin's central question. As introduced above in ‘Background’, Lewontin's focus is not on whether one can do classification, but on what a racial classification conveys about genotype. Repeatedly in his writings and interviews, he conceded the human ability to build classification systems using biological traits like skin pigmentation, hair colour and stature that have genetic components that vary across human groups substantially: ‘No one would mistake a Chinese for a West African or a Finn for an Australian aborigine’ [88, p.111]. The question is whether such race groupings have *taxonomic value* in the sense that they are predictive of *meaningful* differentiation at a *typical genetic locus*. For instance, in his retelling of the result in *Human Diversity*, after granting that loci with large frequency differences between populations like *Duffy Fy* exist, Lewontin stated, ‘The question is, are these large differences typical?’ [88, p.117]. The question was especially of interest for Lewontin because of his concern that visible traits may bias our perception of how much difference exists at the typical genetic locus. With that concern, the focus is naturally on how variation is apportioned per locus.

### (c) Two sides of the same problem

The confusion about the key question does not invalidate the points of Mitton, Smouse and Speilman and Edwards. They were correct that with large numbers of loci, assignment to groups is possible even if the groups are only subtly distinguished (i.e. classification using a genetically informed taxonomy is possible). Even the most subtly differentiated populations become detectably different with enough markers and individuals sampled, making classification possible (see [89] and references within, also [24]). For example, Patterson *et al.* [24] derive an approximate relationship between the number of observed markers, the number of sampled individuals, the level of differentiation in terms and the resultant ability to detect population structure. (These facts resonate for me, given my own experiences detecting subtle gradations of genetic structure in Europe using PCA [23,90].)

The challenge is that two key facts can be true at the same time: an apportionment of variance that implies the differentiation among artificially labelled groups is minuscule at any typical genetic locus does not preclude using all the loci together to identify groupings of individuals.

A perspective article from Anthony Edwards is illuminating here [91]. In the article, he shares this quote by the famed Captain Robert Fitzroy, of the HMS Beagle, who in 1839 wrote: ‘The conclusion to which I have been obliged to come is—that there is far less difference between most nations, or tribes (selecting any two for the comparison), than exists between two individuals who might be chosen out of either one of those nations or tribes; colour and hair alone excepted’.

The quote is compelling here for two reasons. First, there is the similarity between Fitzroy's conclusion and what would come to be understood through genetics via Lewontin's analysis. Second, Edwards does not argue against Fitzroy's interpretation. Edwards treats the apportionment of diversity in humans as a somewhat obvious matter of fact. What he took issue with was the implication taken from Lewontin's paper that one cannot do classification (including building phylogenies) using genetics. Edwards would later say in an interview ‘it is essentially that there are two problems. He [Lewontin] really wasn't going from the variation problem to the phylogenetic problem’ [7, p. 422].

For his part, Lewontin in his work and later writings, made clear that he recognized that some form of classification is possible (e.g. [54,72])—his goal instead was to debate the apportionment of diversity associated with existing classifications of human races. As such, both Lewontin and his apparent critics like Edwards can be understood as addressing two closely related problems, neither actually contradicting the other.

## 4. The long-term legacy

### (a) The apportionment as sound bite

An important antecedent for Edwards' critique, relative to the earlier critiques in the late 1970s, is that by the time Edwards was writing, the apportionment of diversity result had been taken up broadly and become an iconic, politicized and sometimes over-simplified sound bite.

Lewontin's paper became continually more important through time (see [5]). Some of its growing influence was facilitated by Lewontin himself. Lewontin regularly interacted with social scientists and philosophers of science, often hosting visiting scholars in his laboratory. He was active in a public outreach group *Science for the People*. He repeated the results, for instance in his 1974 book *The Genetic Basis of Evolutionary Change* (Richard C. [92]), in a 1982 book titled *Human Diversity* that was part of the public-facing Scientific American Library Series [88], and in his book *Not in Our Genes* [34].

However, beyond Lewontin and the 1972 paper, the empirical fact of the 85%/15% split in diversity within versus between populations grew a life of its own and was amplified in numerous ways. This was in part facilitated, as remarked above, because many other population genetics researchers found similar results, which had the effect of amplifying knowledge of the apportionment of diversity. The highly influential 1981 book *The Mismeasure of Man* by Lewontin's Harvard colleague Stephen J. Gould, cited Lewontin's result in its closing chapter [93]. Another amplifier, though indirect, may have been an influential 1985 paper by philosopher Kwame Anthony Appiah [94,95]. Appiah argued there is ‘no biological basis to race’ and his opening paragraphs reference the apportionment of diversity via a review paper of Nei and Roychoudhury from 1982  [96] (see Carlson & Harris [5], which contains a section on the legacy of Nei and Roychoudhury's work [96]). Given the strong downstream influence of Appiah's work, the 1985 paper may have played a role in coupling the apportionment of diversity with the philosophical position of ‘no biological basis to race’ [95]. By 2001, when the draft human genome sequences were published, the apportionment of diversity result had become widespread as the sound bite ‘there is more genetic variation within populations than between populations’, with the direct ties to Lewontin being obscured (Carlson & Harris [5]).

It was in this milieu that Anthony Edwards was approached by his brother to respond to statements about the lack of a biological basis for human races [7,91]. His concern was that statements circulating at the time seemed to dispel any possibility of using genetics to study population history, a field that Anthony Edwards had helped pioneer with the development of clustering and phylogenetic methods [46,47]. Thus, an initial draft of Edwards' paper was entitled, ‘The Death Of Phylogeny’ and only later, after Edwards traced the origin of the offending statements to Lewontin, was the title changed to be ‘Human Genetic Diversity: Lewontin's Fallacy’ [91].

As an example of a motivating statement, in the 2003 article, Edwards cited a 2001 piece in *Nature* that claimed ‘two random individuals from any one group are almost as different as any two random individuals from the entire world’. Such a statement is true if we inspect a ‘typical’ or random locus in the genome of the two individuals. However, as statements about genome-wide multi-locus similarity, it is necessary to introduce more nuance [97]. In terms of the number of pairwise differences between two individuals, the 85%/15% result of Lewontin implies that two random individuals from a given ancestry will have on average 15% fewer pairwise differences than individuals sampled across the globe. So the statement that the two individuals from one group are ‘almost as different’ is vague but not indefensible. Consider instead the closely related claim that individuals from distinct populations will be more similar than individuals from the same population. This is highly likely when we look at the typical locus in the genome; however, for multi-locus similarity the claim is often going to be wrong. A paper by Witherspoon *et al.* [17] explored the same question empirically. Asking whether individuals from distinct populations will be more similar than individuals from the same population, they found that when focusing on 100 loci the answer is about 20% of the time, but ‘if genetic similarity is measured over many thousands of loci, the answer becomes ‘never’ [17].

These examples stress how it is important to be clear about what units are being inspected—whether it's a single ‘typical’ locus or ‘scoring the whole genome’ in Chakroborty's phrasing (see above). Importantly, regardless of the subtleties that arise in evaluating these statements, Lewontin's key point holds: that human race is ‘skin deep’ in the sense that the apportionment of genetic diversity at a typical locus does not agree with what we see from physical features; as a taxonomic category, race only conveys a small fraction of the total genetic variation in humans.

### (b) Not a silver bullet

In appreciating the insight that Lewontin's result provided, it is important to understand its limits.

The 1972 paper did not convey concretely how traits might vary across groups. Later works by several others extended the implications of Lewontin's work to quantitative traits (see [98,99] and references therein). Interestingly, in simple models, the same apportionment of diversity seen at single loci should hold for strictly *neutrally* varying quantitative traits (i.e. traits whose variants have no pleiotropic fitness effects). This supports Lewontin's view in that, for a species with an apportionment of genetic diversity like that of humans, one should expect neutrally varying traits to not greatly differ between populations either.

That said, elevated differentiation between human groups in genetic variation underlying traits can arise for several reasons: if the loci underlying a trait have experienced geographically variable selective pressures (as seen with skin pigmentation loci), or less obviously, in various cases where there is stabilizing selection on a trait, even when there is a single optimum shared between species (e.g. [100,101]). Overall, the degree to which variable selective pressures have acted on variants underlying human traits is still unknown and is often difficult to understand rigorously using techniques developed to date [101–103].

For some of the most controversial traits of interest (such as behavioural ones), many practising population geneticists think geographically varying selective pressures are unlikely and that any differences (due to selection or otherwise) will be negligible, especially relative to the rich prior knowledge of environmental factors and interactions that can shape such traits (Lewontin was a leader in expressing and arguing this perspective). Others think that differences between groups in some form (selected or otherwise) are more likely to exist than not (e.g, ‘it would be a bad bet to argue’ [79, p. 258]), and we should prepare society to deal with such group differences if they are discovered (reaffirming the widely held ethical position of assigning moral equality to all individuals), as otherwise pseudo-scientific approaches and interpretations would likely lead the discussion of any possible differences.

For Lewontin's part, he seems to have understood the main limitations and the utility of his results [54,72,104]. For instance, both are expressed in his Hitchcock lecture at Berkeley in 2003 [72]. In the lecture, he acknowledges that his work ‘does not prove … that there isn't a gene some place’ that might be important for affecting behaviour and that varies across populations. While admitting this, he stressed defensively that ‘nobody's ever found it’ and ‘there's no reason to think such things exist’. In a frank response to a question about what effect his work may have on those with racial prejudices, he expressed ‘it's not clear it has any effect … I have not proved [racially differentiated genes for IQ] don't exist … .’ He continued, ‘I think data like these in large part, predispose one toward an understanding of the situation, but if you're a hardcore racist they're not going to have any effect at all’.

As Lewontin knew, his result is not a silver bullet that defeats all possible racist positions, but it does set an important prior expectation. On a scientific basis, one can safely expect that the average variant is not substantially differentiated across race groups, and in turn that race is a poor proxy for genotype at any one locus of interest. For any discussion of human variation, this is an important ‘understanding of the situation’.

## 5. Conclusion

After considering the controversies and confusions that have arisen from Lewontin's 1972 [2] result, it is clear the statistical subtleties of this topic certainly contributed to the confusion—at times it seems like Lewontin and critics like Edwards are involved in the proverbial story of the blindfolded individuals describing an elephant where one is feeling the trunk and the other the legs. This conciliatory view is not novel [8,105,106], though hopefully this article helps convey how the apparent disagreements weaken upon inspection.

While there are many intellectual frameworks to understand scientific controversies (e.g. [107]), the legacy of Lewontin's paper [2] brings to mind  a mode of science posited by sociologist of science Aaron Panofsky that he calls ‘misbehaving science’ [108]. In this mode, controversy is ‘persistent’, ‘ungovernable’ and ‘political’ and ‘scientists are confounded to draw the boundaries between politics and science’. And ‘if science is like a machine for resolving controversies, in misbehaving science that machine is broken’.

In the context of paper [2] and its legacy, the idea of ‘misbehaving science’ resonates but is not a perfect description of the situation. On the one hand, the persistent controversy and legacy following [2] seems outside of normal science. For example, the early controversies following the 1972 publication remerged again thirty years later in 2003. The result became a sound bite about race that is sometimes used vaguely or in mistaken ways. The existence of a paper titled ‘Lewontin's Fallacy’ continues to be used, wrongly, in online discussions of race as if Edwards' paper is a sufficient counter-argument to Lewontin's perspective on the typical variant. Rarely do normal scientific results have such a complex fate and political life. On the other hand, upon close inspection, the key scientific controversy falls away, and this has been appreciated for a long time (see [105,106] and [8]). Science has ‘behaved’ itself in a sense, even though the political controversy and confusions about the implications of the science have been persistent.

Finally, in reflecting on the legacy of Lewontin's work, I hope this narrative has made clear some of the enduring and positive aspects of Lewontin's work. To summarize, his work helps us understand that the typical locus in the human genome is not as differentiated as one might guess from looking at our external features. This does not imply that classification using multi-locus genomic data is not possible; conversely the ability to use multi-locus data to study how individuals genetically relate to one another does not invalidate Lewontin's result and main conclusion. Fifty years after the publication, Lewontin's key empirical claim still continues to hold true. We continue to see that for a typical variant, most variance is found within human groups and little is found between them. This fact has never been a ‘silver bullet’ that ends all discussion of genetics and race. Yet, as a critique of race as a useful classification system, it still deepens our understanding of human variation, still challenges simple interpretations about human differences, and helps affirm the willful choice to see past the superficial in how we as humans relate to one another.

## Data Availability

This article has no additional data.
